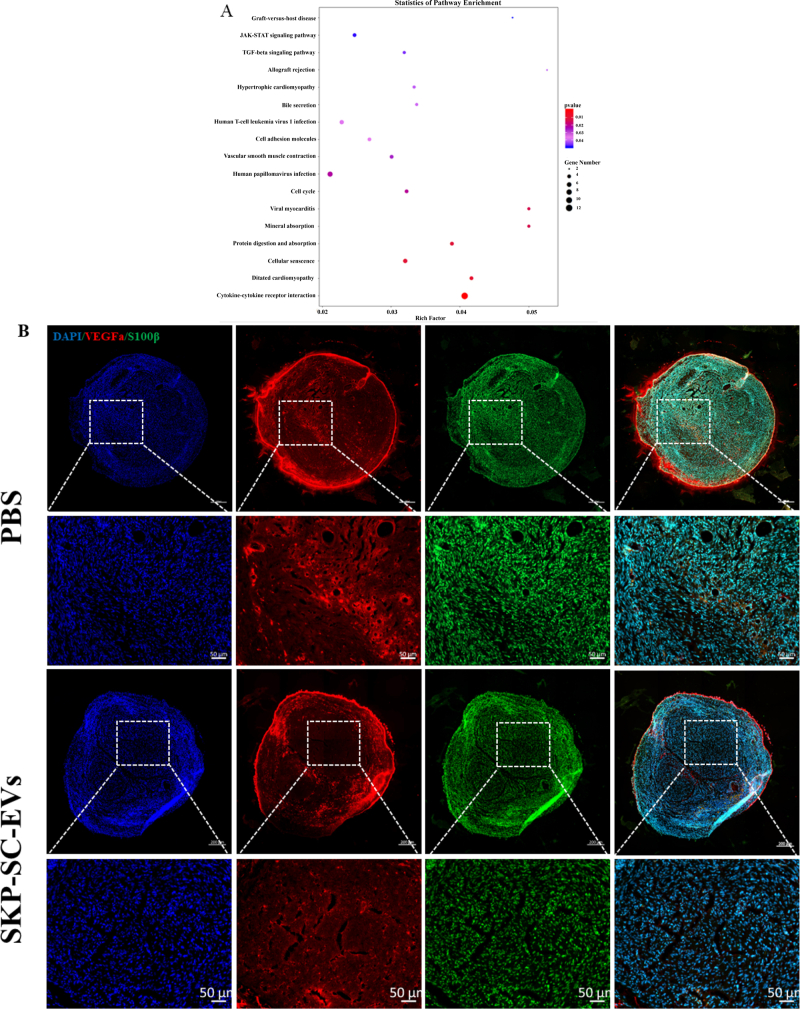# Angiogenesis-promoting effect of SKP-SC-EVs-derived miRNA-30a-5p in peripheral nerve regeneration by targeting LIF and ANGPT2

**DOI:** 10.1016/j.jbc.2024.108146

**Published:** 2024-12-26

**Authors:** Mi Shen, Xinli Ye, Qiang Zhou, Mengru Zheng, Mingzhi Du, Lijuan Wang, Meng Cong, Chang Liu, Chunyan Deng, Zhen Xu, Yu Wang, Jiyu Li, Min Feng, Yujiao Ye, Shuyu Zhang, Wenqing Xu, Yi Lu, Junjie Kong, Jiahuan Gong, Yingjie Xia, Jinhua Gu, Huimin Xie, Qianru He, Qi Zhang, Hualin Sun, Xingjun Liu, Leilei Gong, Miaomei Yu, Xiaosong Gu, Jian Zhao, Ning Zhang, Fei Ding, Songlin Zhou

**Affiliations:** 1 Key Laboratory of Neuroregeneration of Jiangsu and Ministry of Education, Co-innovation Center of Neuroregeneration, NMPA Key Laboratory for Research and Evaluation of Tissue Engineering Technology Products, Nantong University, Nantong, Jiangsu, China; 2 Department of Clinical Medical Research Center, The Affiliated Suqian First People's Hospital of Nanjing Medical University, Jiangsu Province, China; 3 Department of Orthopedic Oncology, Second Affiliated Hospital of Naval Medical University, Shanghai, China; 4 Medical School of Nantong University, Nantong, Jiangsu, China; 5 Nantong Institute of Genetics and Reproductive Medicine, Affiliated Maternity & Child Healthcare Hospital of Nantong University, Nantong, China; 6 The Affiliated Nantong Stomatological Hospital of Nantong University, Nantong, China; 7 School of Pharmacy, Nantong University, Nantong, Jiangsu Province, China; 8 Clinical Medical Research Center, The Third Affiliated Hospital of Soochow University, Changzhou, Jiangsu, China; 9 Jiading Branch of Shanghai General Hospital, Shanghai Jiao Tong University School of Medicine, Shanghai, China

**Keywords:** skin precursor-derived Schwann cells, extracellular vesicles, peripheral nerve injury, angiogenesis, miR-30a-5p, ANGPT2, LIF

## Abstract

Ischemia and hypoxia caused by vascular injury intensify nerve damage. Skin precursor-derived Schwann cells have demonstrated an accelerated *in vivo* prevascularization of tissue-engineered nerves. Furthermore, extracellular vesicles from skin precursor-derived Schwann cells (SKP-SC-EVs) show the potential in aiding peripheral nerve regeneration. Nonetheless, the capacity of SKP-SC-EVs to facilitate nerve repair *via* angiogenesis remains uncertain. This study observed that SKP-SC-EVs significantly enhanced angiogenesis, evidenced by increased transparency of the tissue-engineered nerve graft and ultrasonic blood flow imaging. *In vitro* experiments confirmed that SKP-SC-EVs promote the proliferation, migration, and tube formation of human umbilical vein endothelial cells, a standard model for assessing angiogenic potential. Additionally, a comprehensive miRNA expression profile of SKP-SC-EVs was performed, leading to the identification of potential candidates through functional experiments. Among these, miR-30a-5p emerged as a significant candidate, demonstrating remarkable proangiogenic effects both *in vivo* and *in vitro*, akin to the effects of SKP-SC-EVs. Furthermore, luciferase reporter assay and functional experiments revealed that miR-30a-5p in SKP-SC-EVs promotes angiogenesis by targeting ANGPT2 and LIF without sufficient VEGFa. Thus, the enrichment of miR-30a-5p in SKP-SC-EVs indicates its pivotal role as a regulator of angiogenesis, presenting a promising avenue for cell-free treatment of peripheral nerve injury.

Peripheral nerve injury (PNI) can result in secondary injuries and even disability due to factors such as crush or traction trauma and ischemia (1). The sciatic nerve is the most commonly affected nerve in lower limb injuries (2). Nerve repair and regeneration following PNI occur at slow rates and are regulated by various processes, including immune cell infiltration, Schwann cell (SC) phenotype transformation, and the regeneration of axons and nerve vessels (3, 4, 5, 6). Prolonged denervation can lead to changes in end organs similar to those in muscle atrophy, ultimately preventing complete recovery (7). Limited oxygen diffusion creates an insufficient blood supply, which is a significant bottleneck for peripheral nerve defects (8). Although angiogenesis research has primarily focused on tumors, its relationship with tissue regeneration has gained increasing attention in recent years (9, 10). Blood vessels have been shown to guide SC-mediated peripheral nerve regeneration, highlighting the direct link between angiogenesis and nerve regeneration (11). Stat3, Ephb3, and Cdc42 have been identified as key regulatory molecules in peripheral nerve regeneration and angiogenesis, indicating that these two processes are activated on the first day after injury and work cooperatively throughout the regeneration process (12).

Despite extensive research on PNI and regeneration, reliable treatments ensuring full functional recovery remain elusive (13). Current medications exhibit mediocre effectiveness, primarily providing pain relief without significantly accelerating nerve regeneration or functional recovery in severe cases. Several phytochemicals show promise in expediting recovery post-PNI, but their dose dependence and toxicity levels require assessment before advancing to preclinical and clinical trials (14). The gold standard for treating peripheral nerve defects is nerve autografting; yet, it faces limitations such as limited donor nerve availability, unsatisfactory outcomes, and the potential for neuroma and donor site lesions, diminishing its applicability (15). Allogeneic nerve grafts, developed to address donor shortages and secondary damage, are susceptible to immune rejection and may still result in neuroma formation (16). Nerve conduits present an alternative, eliminating the need for donor nerves and associated donor site morbidity while preventing surrounding tissues from invading the space between stumps (17, 18). Cell therapies have proven effective in overcoming slow nerve regeneration and inadequately filled large gaps. SCs, pivotal in axonal regeneration, have shown considerable potential in preclinical experiments. However, their limitations, including challenging harvesting, slow expansion in culture, and high immunogenicity, reduce their effectiveness in clinical studies. Thus, the search for alternative cells is imperative. Numerous promising animal studies indicate that stem/progenitor cell transplantation can restore some degree of injured nerve function and has become a viable cell source to replace SCs in peripheral nerve regeneration (19, 20, 21). As a nonethical stem cell source, skin-derived precursors (SKPs) offer highly accessible myelin cells, capable of differentiating into SCs after induction and demonstrating the ability for myelin formation *in vitro*.

While the survival rate of SKP-SC cells transplanted into the damaged site *in vivo* is low, they significantly promote regeneration following PNI (21, 22). Research has shown that Schwann cells derived from skin precursors accelerate *in vivo* prevascularization of tissue-engineered nerves. The therapeutic efficacy of mesenchymal stem cells (MSCs) is mainly attributed to their paracrine effects, and using MSC-derived extracellular vesicles offers substantial advantages over live cell counterparts (23). Extracellular vesicles (EVs), membranous particles facilitating paracrine communication, exhibit low immunogenicity and transfer nucleic acids, proteins, and lipids between cells (24). These vesicles can be selectively absorbed by other cells, modulating various biological processes (25, 26). Consequently, EVs can effectively circumvent the challenges associated with stem cell therapy. MSC-derived extracellular vesicles from various sources enhance PNI recovery and present novel opportunities for nerve injury repair by promoting neurovascular regeneration (26, 27).

SKP-SC-EVs enhance the activity and regeneration of both motor and sensory neurons, offering a promising novel strategy for PNI treatment (28, 29). This study hypothesized that SKP-SC-EVs could also positively influence nerve repair after PNI by promoting angiogenesis. To test this hypothesis, experiments were conducted to observe the effects of SKP-SC-EVs on endothelial cells and angiogenesis post-PNI. Screening 14 miRNAs in SKP-SC-EVs identified miR-30a-5p as exhibiting notable proangiogenic properties *in vivo* and *in vitro*, mirroring the effects of SKP-SC-EVs. Luciferase reporter assay and functional experiments revealed that miR-30a-5p promotes angiogenesis by targeting angiopoietin-2 (ANGPT2) and LIF. In summary, SKP-SC-EVs and miR-30a-5p may serve as pivotal regulators of angiogenesis in PNI treatment.

## Results

### SKP-SC-EVs could promote regeneration of sciatic nerve and recovery of sensory function in rats by promoting angiogenesis

**Fig. S1:**
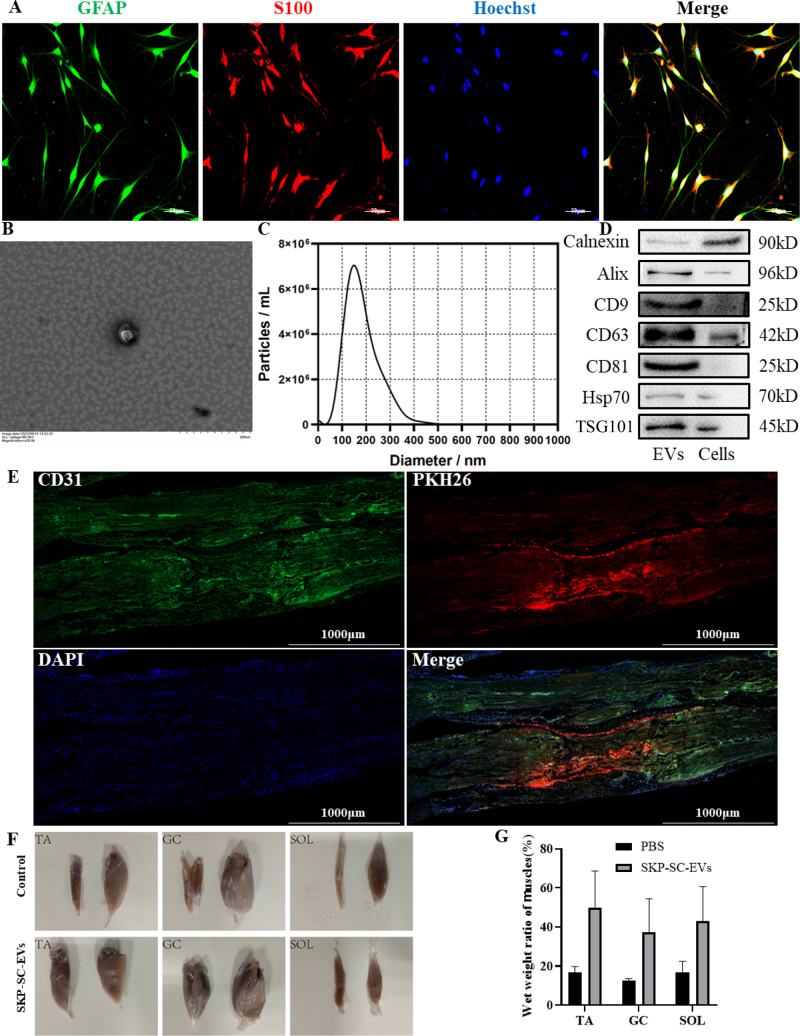


SKP-SCs and SKP-SC-EVs were identified using established methods (30). SKP-SCs displayed a typical bipolar spindle-shaped morphology and grew closely together. Immunofluorescence analysis showed positive expression of SC markers S100 and glial fibrillary acidic protein (GFAP) (Fig. S1*A*). Transmission electron microscopy revealed the sunken cup-shaped structure of EVs (Fig. S1*B*). Nanoparticle tracking analysis indicated a peak particle size of 169.6 nm for EVs, with a concentration of approximately 1.1 × 10^11^ particles/ml (Fig. S1*C*). Western blot analysis demonstrated high expression of EV markers CD9, CD63, CD81, Hsp70, and TSG101, while endoplasmic reticulum protein calnexin showed low expression. This protein expression pattern in SKP-SCs contrasted with that in EVs (Fig. S1*D*). Additionally, EV internalization in the sciatic nerve epineurium was confirmed (Fig. S1*E*).

**Figure 1:**
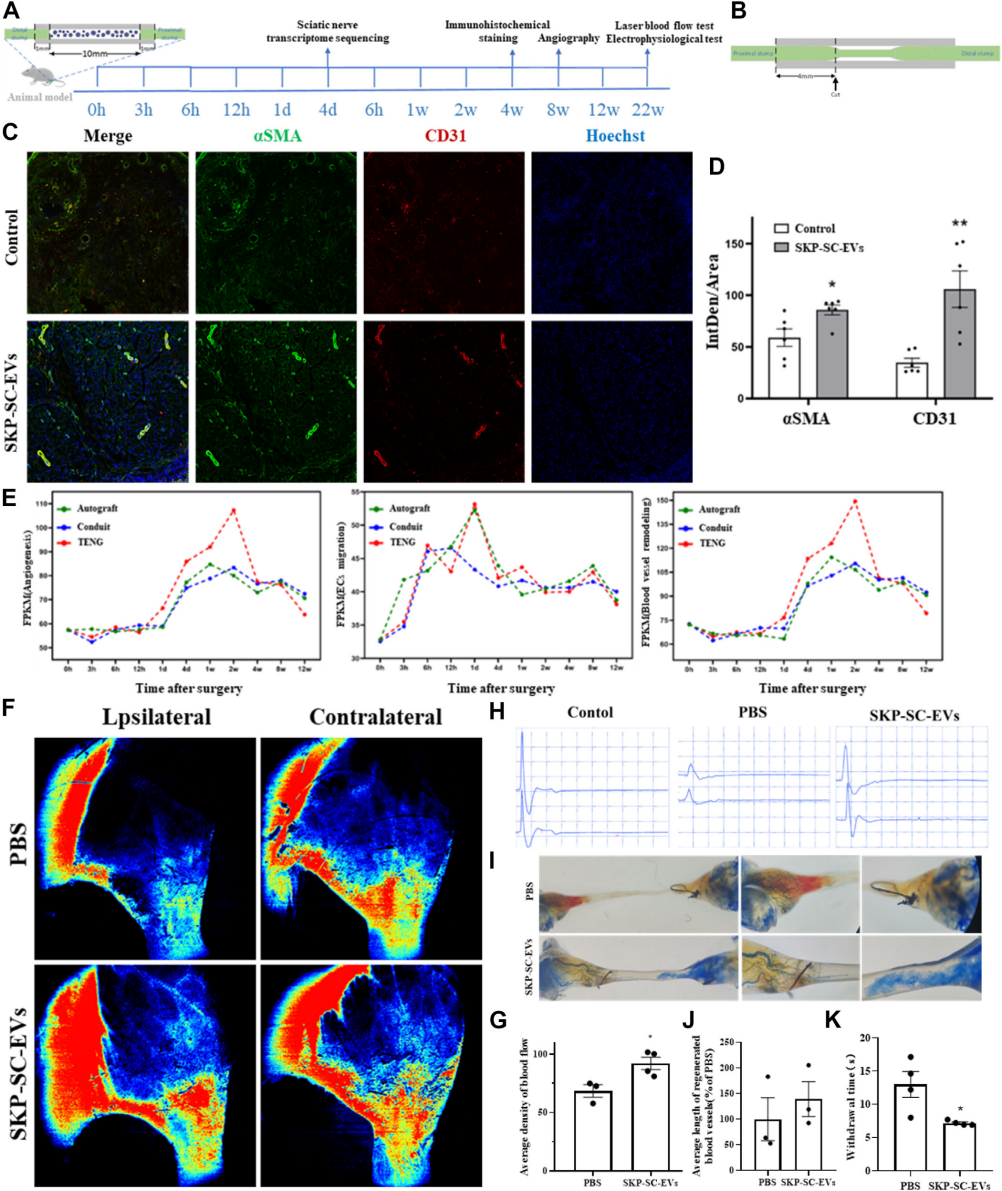
**SKP-SC-EVs facilitate sciatic nerve regeneration and enhance sensory recovery in rats by stimulating angiogenesis**. *A*, the posterior bridge model of sciatic nerve defect, transcriptome sequencing, and the follow-up test time point were established. *B*, frozen sections of the sciatic nerve and graft in rats were examined. *C*, immunofluorescence staining of the rat sciatic nerve revealed higher average fluorescence intensity of CD31 (*red*) and α-SMA (*green*) in the TENG group than in the Conduit group. Additionally, the TENG group exhibited more distinct vascular structures—the scale bar represents 150 μm. *D*, statistical representation of the mean immunofluorescence intensity of the rat sciatic nerve was conducted. ∗*p* < 0.05, ∗∗*p* < 0.01 *versus* control, assessed by Student's *t* test, n = 4 (one rat died from anesthesia and was excluded). *E*, trends in gene expression related to local microenvironment angiogenesis during sciatic nerve defect repair, trends in chemotaxis-related gene expression in local microenvironment endothelial cells during sciatic nerve defect repair, and changes in gene expression related to local microenvironment vascular remodeling during sciatic nerve defect repair were assessed. *F*, blood flow distribution of the injured hind limb was detected by laser flow meter. *G*, results demonstrate a significantly higher blood perfusion rate in the EV group than in the vehicle group. ∗*p* < 0.05 *versus* control group, assessed using Student's *t* test, n = 5. *H*, the electrical conduction function of rats on the injured side was evaluated. Profiling the amplitude of CMAP or MCV in the EVs group revealed significantly higher values than in the vehicle group. *I*, light microscopy image of an angiogram. Contrast material filled the microvessels (*blue*) at the proximal and distal ends of the ischium. *J*, the average length of regenerated blood vessels was measured. Student's *t* test, n = 3. *K*, thermal sensitivity was assessed using a plantar test. The withdrawal time of rats in the SKP-SC-EVs group was significantly lower than that in the PBS group. ∗*p* < 0.05 *versus* control, assessed using Student's *t* test, n = 4. Data are expressed as mean ± SEM. SKP-SC-EVs, extracellular vesicles from skin precursor-derived Schwann cells; CMAP, compound muscle action potential; MCV, motor nerve conduction velocity; TENG, tissue-engineered nerve graft; α-SMA, alpha smooth muscle actin.

A tissue-engineered nerve graft (TENG) was fabricated within a silicone conduit, loaded with either the vehicle or EVs mixed with Matrigel, to bridge a rat sciatic nerve defect (Fig. 1*A*). After 6 months of treatment, the nerve bridge segment was sectioned using a cryostat (Fig. 1*B*) and subjected to immunofluorescence staining with angiogenesis markers CD31 and alpha smooth muscle actin. Enhanced angiogenesis was observed in the SKP-SC-EVs group (Fig. 1, *C* and *D*). Weighted gene coexpression network analysis results also showed elevated scores for angiogenesis, endothelial cell migration, and blood vessel remodeling in the gene expression profiles of the EVs group (Fig. 1*E*). Laser flowmeter assessment revealed a significantly higher blood perfusion rate in the EVs group compared to the vehicle group (Fig. 1, *F* and *G*). The EVs group also exhibited significantly higher compound muscle action potential (CMAP) and motor nerve conduction velocity amplitudes, indicating improved electrical conduction in injured nerves (Fig. 1*H*). Following dehydration using a glycerin gradient, the nerve stumps and connective tissues became transparent, revealing numerous blood vessels filled with a blue contrast agent under light microscopy. Extensive vascular ingrowth was observed in the tissue-engineered nerves of the EVs group from both proximal and distal ends (Fig. 1, *I* and *J*). The plantar test indicated a significantly shorter withdrawal time for rats in the SKP-SC-EVs group compared to the PBS group (Fig. 1*K*). Additionally, the EVs group displayed thicker regenerated nerves and a significantly higher muscle wet-weight ratio compared to the vehicle group (Fig. 1, *F* and *G*). These findings suggest that SKP-SC-EVs promote sciatic nerve regeneration and sensory function recovery by enhancing angiogenesis in rats.

### SKP-SC-EVs can be internalized by HUVECs and enhance cell viability, migration, and tubulogenesis

**Figure 2:**
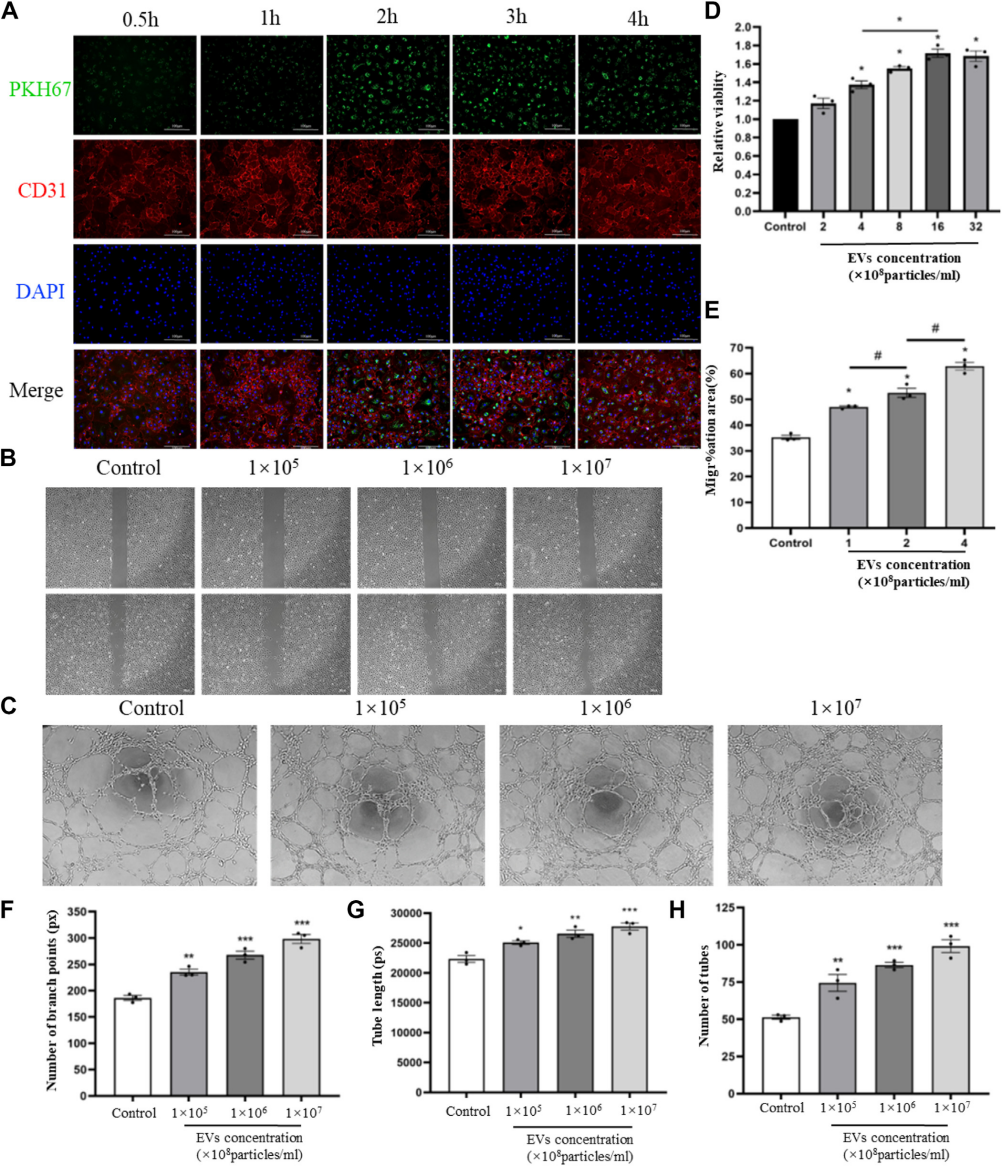
**SKP-SC-EVs can be internalized by HUVECs, leading to enhanced cell viability, migration**, **and tubulogenesis**. *A*, after incubation for different periods, PKH67 antibody-labeled EVs (*green*) were observed in the cytoplasm of CD31-labeled HUVECs (*red*). The signal intensity was positively correlated with time. The scale bar represents 100 μm. *B*, CCK8 assay was conducted to analyze EVs' enhancement of HUVECs viability statistically. ∗*p* < 0.05 *versus* control, assessed by one-way analysis of variance (ANOVA), n = 3. *C*, light microscopy images of wound healing assay in which EVs promoted HUVEC migration in a dose-dependent manner. The scale bar represents 200 μm. *D*, statistical plot of wound healing experiments in which SKP-SC-EVs promoted HUVECs migration. ∗*p* < 0.05 *versus* control group, one-way analysis of variance, n = 3. *E*, matrix gel methodology was used to study SKP-SC-EVs' promotion of HUVECs tube formation. The scale bar represents 200 μm. *F*–*H*, statistical diagrams representing the results of the matrigel method for SKP-SC-EVs' promotion of HUVECs tube formation were generated. The number of branches (*F*), tube length (*G*), and tube number within the tubular structure (*H*) were quantified. ∗*p* < 0.05, ∗∗*p* < 0.01, ∗∗∗*p* < 0.005 *versus* control, assessed by one-way ANOVA, n = 3. Data are expressed as mean ± SEM. SKP-SC-EVs, extracellular vesicles from skin precursor-derived Schwann cells; CCK8, Cell Counting Kit-8; HUVEC, human umbilical vein endothelial cell.

After labeling human umbilical vein endothelial cells (HUVECs) with PKH67-labeled EVs, immunofluorescence assays were used to assess EV internalization by HUVECs. A significant positive PKH67 signal was observed within the cell cytoplasm, intensifying over time, indicating successful EV internalization (Fig. 2*A*). Subsequent Cell Counting Kit-8 (CCK8) and wound healing assays revealed a dose-dependent increase in HUVEC viability (Fig. 2*B*) and migration ability (Fig. 2, *C* and *D*) in response to EV treatment. The tube formation assay measured the number of branches, tube length, and tube count in the tubular structure, with representative images presented in Fig. 2*E*. Compared to the control group, EVs significantly enhanced tube-forming ability, with statistical results showing a dose-dependent trend (Fig. 2, *F*–*H*).

### miR-30a-5p was abundant in SKP-SC-EVS and transferred to HUVECs through EVs

**Figure 3:**
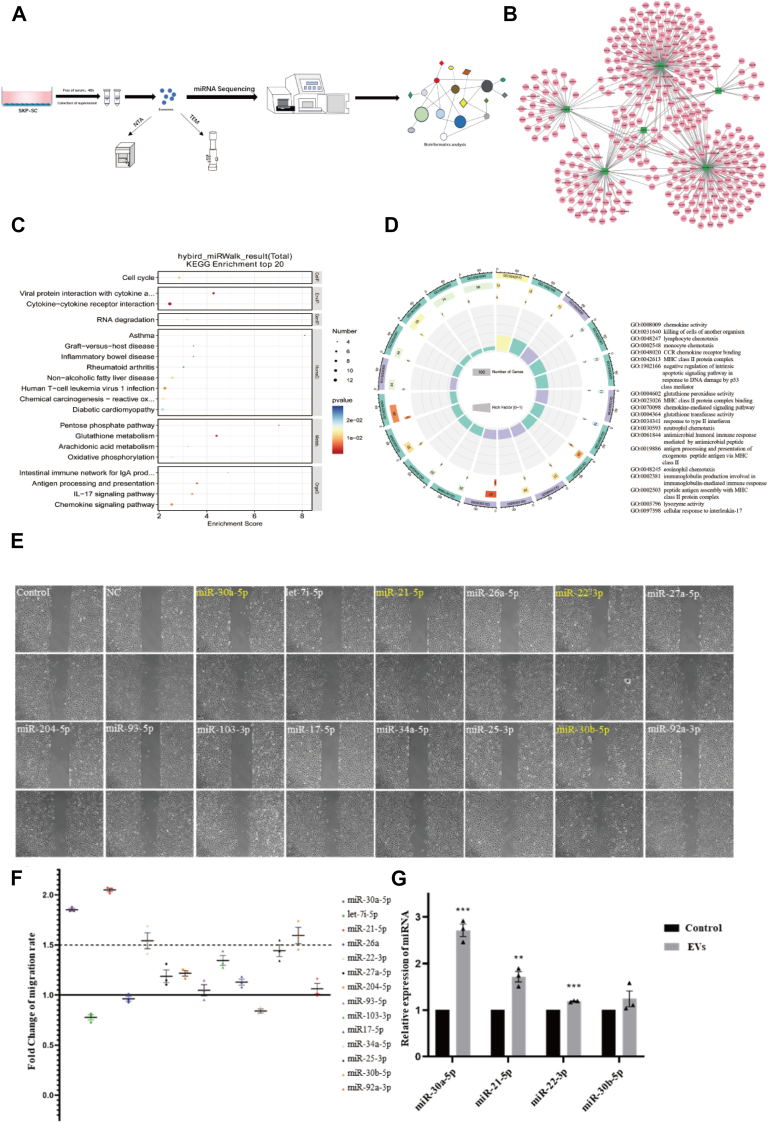
**miR-30a-5p was highly abundant in SKP-SC-EVs and transferred to HUVECs using EVs**. *A*, flow chart outlining the process of EV extraction and miRNA sequencing. *B*, network diagram illustrating the target genes of the miRNA. *C*, target gene enrichment at the intersection of the Hybrid and miRWalk databases. *D*, Gene Ontology (GO) enrichment circle depicting different classifications and corresponding gene counts. The first *circle* serves as the coordinate ruler for the gene numbers. Various colors represent distinct classifications. The *second circle* displays the taxon count within the background genes, with longer bars indicating more genes. *Reddish hues* denote smaller values, while *bluish hues* signify larger values. The *fourth circle* shows Rich Factor values for each category (the number of foreground genes divided by the number of background genes in that category); background auxiliary lines represent 0.2 per small cell. *E*, light microscopic view of wound healing, demonstrating that miR-30a-5p promotes migration of HUVECs. The scale bar represents 200 μm. *F*, fold difference map depicting the enhancement of HUVECs migration by miR-30a-5p (with NC as the reference). *G*, qPCR validation plot illustrating miRNA expression levels in HUVECs after EV treatment. ∗∗*p* < 0.01, ∗∗∗*p* < 0.005 *versus* control, assessed by Student's *t* test, n = 3. Data are expressed as mean ± SEM. SKP-SC-EVs, extracellular vesicles from skin precursor-derived Schwann cells; HUVEC, human umbilical vein endothelial cell; qPCR, quantitative PCR.

To explore the mechanisms underlying EV-mediated angiogenesis, the study hypothesized that miRNAs play pivotal roles, given the established proangiogenic effects of EVs *in vitro*. EVs were sequenced to identify miRNA profiles (Fig. 3*A*). Network analysis suggested potential targets of highly abundant miRNAs (Fig. 3*B*). For instance, miR-30c-2-3p, associated with gastric cancer, can suppress malignant progression by inhibiting ARHGAP11A (31). Enrichment analysis revealed pathways and factors such as the cell cycle, chemokines, lysosomal activity, and interleukin-17 among the top 20 Gene Ontology (GO) and Kyoto Encyclopedia of Genes and Genomes terms (32). Notably, the IL-17A-HIF1α axis drives glycolysis in wound front epithelia, and loss of interleukin-17 impairs wound repair by epithelial cells (Fig. 3, *C* and *D*). Based on these data and prior studies on miRNAs influencing cell migration, 14 miRNAs were selected for further investigation. Transfecting these 14 miRNAs into HUVECs revealed that miR-30a-5p, miR-21-5p, miR-22-3p, and miR-30b-5p significantly promoted migration (fold change > 1.5; Fig. 3, *E* and *F*). Subsequently, quantitative real-time polymerase chain reaction (qRT-PCR) determined the expression changes of these miRNAs in HUVECs after EV treatment, showing miR-30a-5p had the most significant upregulation (Fig. 3*G*).

### miR-30a-5p promoted angiogenesis *in vitro* and *in vivo*

**Figure 4:**
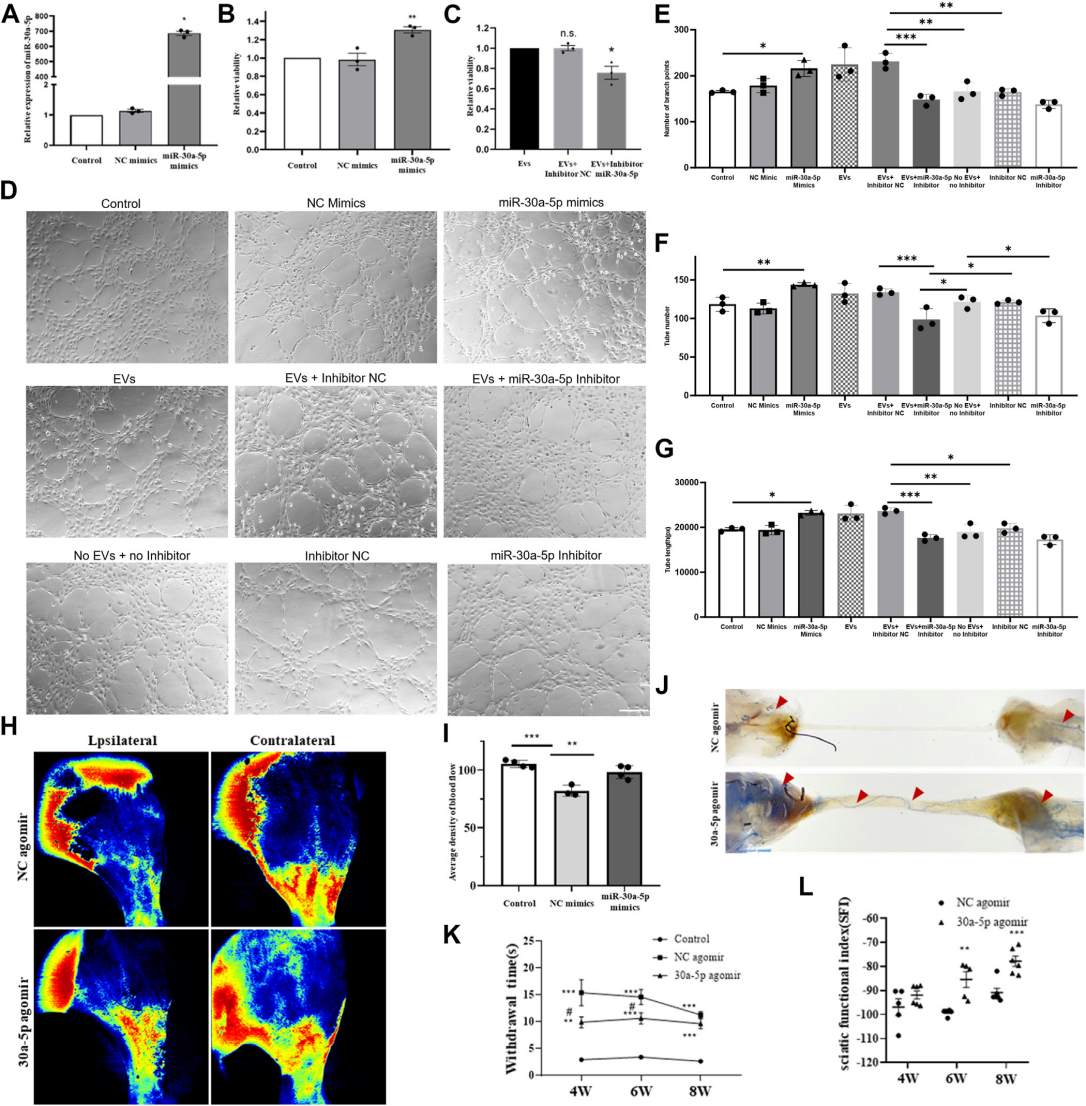
**miR-30a-5p exhibited proangiogenic effects both *in vitro* and *in vivo***. *A*, statistical plot confirming the transfection efficiency of miR-30a-5p using qPCR. ∗*p* < 0.05 *versus* control, assessed using one-way analysis of variance (ANOVA), n = 3. *B*, CCK8 assay graph displaying the promotion of HUVECs viability by miR-30a-5p. ∗*p* < 0.05 *versus* control, assessed using one-way ANOVA, n = 3. *C*, knockdown of miR-30a-5p countered the proviability influence of SKP-SC-EVs on HUVECs, as shown in the CCK8 statistical diagram. ∗*p* < 0.05 *versus* EVs, assessed by one-way ANOVA, n = 3. *D*, light microscopy image depicting the matrigel method used to evaluate miR-30a-5p′s promotion of HUVECs tube formation, the inhibition effect of SKP-SC-EVs tube formation through miR-30a-5p knockdown and the inhibition of miR-30a-5p′s influence of HUVECs tube formation without EVs. The scale bar represents 100 μm. The statistical diagrams illustrating the number of branches (*E*), tube number (*F*), and tube length (*G*) of HUVECs. ∗*p* < 0.05, ∗∗*p* < 0.01, ∗∗∗*p* < 0.005, assessed by one-way ANOVA, n = 3. *H*, laser flowmeter detection of blood flow distribution in the injured hind limb. The blood perfusion rate was significantly higher in the miR-30a-5p mimic group compared to the solvent group. *I*, statistical plot representing the mean blood flow density. ∗∗*p* < 0.01, ∗∗∗*p* < 0.005 versus control, assessed by Student's *t* test, n = 4 (one rat died from anesthesia and was excluded). *J*, light microscopy image of an angiogram. Contrast material filled the microvessels (*blue*) at the proximal and distal ends of the ischium. In the miR-30a-5p group, many blood vessels had grown into the tissue-engineered nerves, even passing through the entire TENG. *K*, thermal sensitivity test results from the plantar test show that rats in the miR-30a-5p mimic group exhibited significantly lower exit times than those in the miR-NC group. ∗∗*p* < 0.01, ∗∗∗*p* < 0.005 *versus* control, #*p* < 0.05 *versus* NC agomir, assessed by one-way ANOVA. (n = 3). *L*, statistical plot representing the sciatic nerve function index (SFI). The SFI of the miR-30a-5p mimic group was higher than that of the miR-NC group. ∗∗*p* < 0.01, ∗∗∗*p* < 0.005 *versus* NC agomir, assessed by one-way ANOVA. Data are expressed as mean ± SEM. (n = 5). SKP-SC-EVs, extracellular vesicles from skin precursor-derived Schwann cells; CCK8, Cell Counting Kit-8; HUVEC, human umbilical vein endothelial cell; qPCR, quantitative PCR; TENG, tissue-engineered nerve graft.

**Fig. S2:**
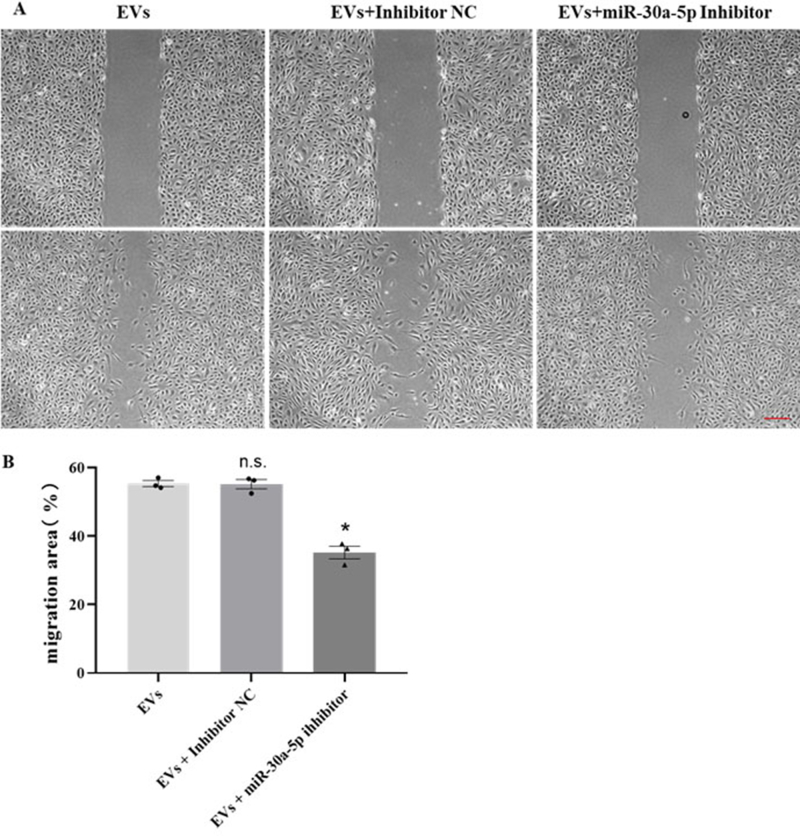


To determine whether miR-30a-5p regulates angiogenesis and acts as a biological messenger between EVs and HUVECs, cells transfected with mimics underwent qRT-PCR, vitality, and tubulogenesis assays. miR-30a-5p expression significantly increased in HUVECs transfected with miR-30a-5p mimics (Fig. 4*A*). CCK8 assay results showed that miR-30a-5p significantly enhanced HUVEC activity, while a miR-30a-5p inhibitor added to EVs significantly reduced HUVEC activity (Fig. 4, *B* and *C*). The effect of miR-30a-5p on tubulogenesis was assessed next. Compared to the miR-NC group, the miR-30a-5p group displayed significant increases in the number of branches, tube count, and tubular structure length (Fig. 4, *D–G*). The miR-30a-5p inhibitor nullified the EV-induced enhancement of HUVEC migration (Fig. S2, *A*–*B*) and tube formation (Fig. 4, *D*–*G*) and also abrogated HUVEC tube formation without EVs (Fig. 4, *D*–*G*). Compared to the no EVs + no inhibitor group, the EVs + NC inhibitor group displayed significant increases in the number of branches, tube count, and tubular structure length and this enhancement effect of EVs could be entirely inhibited by the miR-30a-5p inhibitor (Fig. 4, *D*–*G*). In rats with sciatic nerve defects, blood flow distribution was assessed in injured hind limbs treated with TENG loaded with miR-30a-5p mimic mixed with Matrigel. The blood perfusion rate in the miR-30a-5p mimic group was significantly higher than in the vehicle group (Fig. 4, *H* and *I*). Microvessels filled with a blue contrast agent indicated abundant blood vessel infiltration across the entire TENG in the miR-30a-5p groups (Fig. 4*J*). Plantar test results showed a significantly shorter withdrawal time for rats in the miR-30a-5p mimic group compared to the miR-NC group (Fig. 4*K*). Additionally, the SFI in the miR-30a-5p mimic group exceeded that of the miR-NC group (Fig. 4*L*). Lastly, CD31 staining showed enhanced angiogenesis in response to miR-30a-5p (Fig. S2*C*).

### SKP-SC-EV-derived miR-30a-5p regulates ANGPT2 and LIF by directly targeting the 3′-UTR

**Figure 5:**
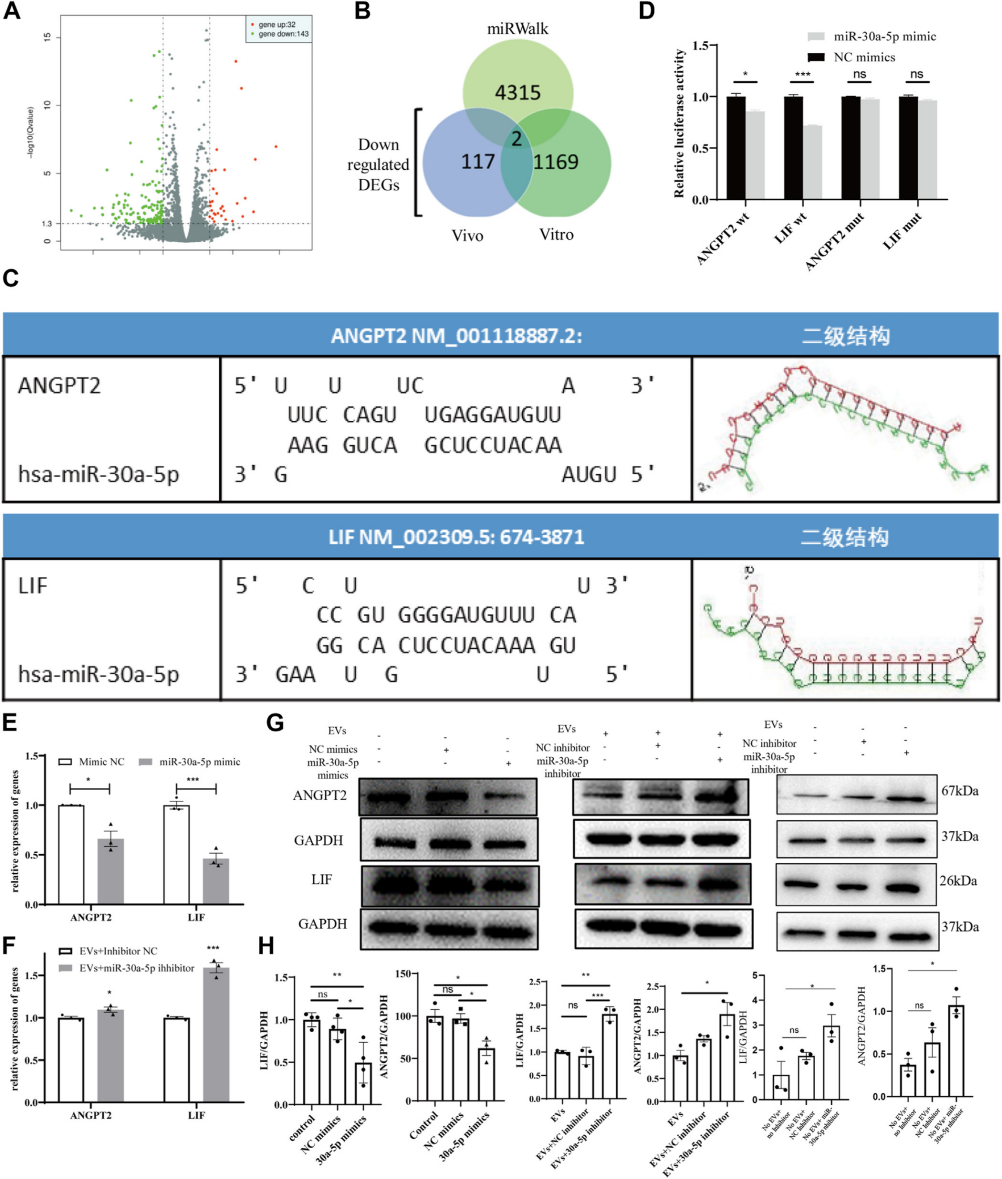
**SKP-SC-EVs**-**derived miR-30a-5p directly regulates ANGPT2 and LIF by targeting their 3′-UTR regions**. *A*, map illustrating differentially expressed genes. Following miR-30a-5p transfection in HUVECs, transcriptome sequencing revealed 32 upregulated and 143 downregulated genes. *B*, venn diagram depicting the intersection between the database and differentially expressed genes. The genes downregulated in transcriptome sequencing were cross-referenced with miRWalk, both *in vitro* and *in vivo*, resulting in the identification of two target genes (ANGPT2 and LIF). *C*, schematic representation of the binding sites of ANGPT2 (*top*), LIF (*bottom*), and miR-30a-5p. *D*, luciferase assay results. ∗*p* < 0.05, ∗∗∗*p* < 0.005 *versus* NC mimics, assessed by Student's *t* test. *E*, qRT-PCR verification of changes in gene expression after miR-30a-5p mimic transfection. ∗*p* < 0.05, ∗∗∗*p* < 0.005 *versus* Mimic NC, assessed by Student's *t* test, n = 3. *F*, qPCR validation of changes in gene expression after miR-30a-5p inhibitor transfection and EV treatment. ∗*p* < 0.05, ∗∗∗*p* < 0.005 *versus* Mimic NC, assessed using Student's *t* test, n = 3. *G*, Western blot verification of changes in protein expression after miR-30a-5p mimic transfection or miR-30a-5p inhibitor transfection followed by EV treatment or miR-30a-5p inhibitor transfection without EV treatment. *H*, statistical representation of LIF and ANGPT2 protein expression content. ∗*p* < 0.05, ∗∗*p* < 0.01, ∗∗∗*p* < 0.005 *versus* control or EVs, assessed by one-way ANOVA, n = 3. Data are expressed as mean ± SEM. ANGPT2, angiopoietin-2; HUVEC, human umbilical vein endothelial cell; qRT-PCR, quantitative RT-PCR; SKP-SC-EVs, extracellular vesicles from skin precursor-derived Schwann cells.

To identify the target genes of miR-30a-5p, transcriptome sequencing on HUVECs transfected with miR-30a-5p revealed differentially expressed genes (Fig. 5*A*). The top enriched pathways included cytokine-cytokine receptor interaction, cell cycle, cell adhesion molecules, cellular senescence, Jak-Stat signaling, and vascular smooth muscle contraction, indicating miR-30a-5p′s influence in HUVECs (Fig. S3*A*). By integrating downregulated genes from the tissue-engineered nerve graft filled with EVs, cells transfected with the miR-30a-5p mimic, and miRWalk-predicted target genes, ANGPT2 and LIF emerged as target genes (Fig. 5*B*). Online prediction tools revealed miR-30a-5p binding sites on the 3′-UTR regions of ANGPT2 and LIF (Fig. 5*C*). In a luciferase reporter assay, cotransfection of pmirGLO-LIF/ANGPT2 3′-UTR and miR-30a-5p mimic into HEK293 cells resulted in significantly lower luciferase activity in cells with wt-LIF/ANGPT2 plasmid compared to the miR-NC group. Conversely, no significant change in luciferase activity was observed in cells with mut-LIF/ANGPT2 plasmid between the miR-30a-5p and miR-NC groups (Fig. 5*D*). Additionally, qRT-PCR and Western blot analyses showed decreased ANGPT2 and LIF expression levels in the miR-30a-5p mimic group compared to the miR-NC group. HUVECs treated with SKP-SC-EVs and transfected with the miR-30a-5p inhibitor exhibited increased expression levels in the EVs + miR-30a-5p inhibitor group compared to the EVs group, and HUVECs treated with the miR-30a-5p inhibitor alone showed higher expression levels compared to the NC inhibitor group (Fig. 5, *E*–*H*).

### ANGPT2/LIF downregulation promotes viability, migration, and tubulogenesis of HUVECs

**Figure 6:**
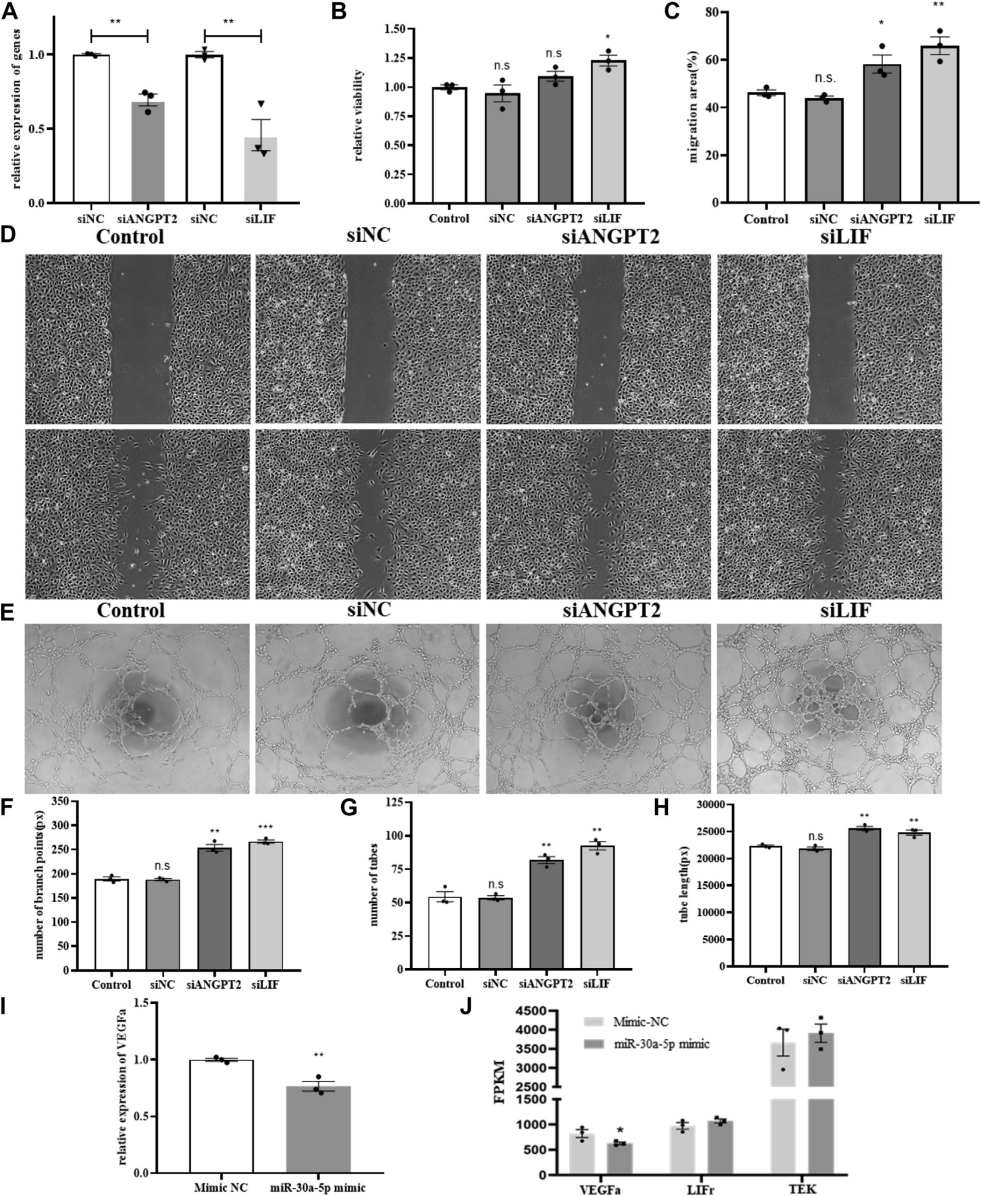
**Downregulation of ANGPT2/LIF enhances the viability, migration, and tubulogenesis of HUVECs**. *A*, statistical plots confirming siANGPT2 and siLIF transfection efficiency using qPCR. ∗∗*p* < 0.01 *versus* siNC, assessed by Student's *t* test, n = 3. *B*, CCK8 statistical plot demonstrating increased HUVECs viability after LIF knockdown. ∗∗*p* < 0.01 *versus* control, assessed by one-way ANOVA, n = 3. *C*, statistical representation of wound healing indicating that knockdown of ANGPT2/LIF promotes HUVECs migration. ∗*p* < 0.05, ∗∗*p* < 0.01 *versus* control, assessed by one-way ANOVA, n = 3. *D*, light microscopic view of wound healing, demonstrating that ANGPT2/LIF knockdown enhances HUVECs migration. The scale bar represents 200 μm. *E*, light microscopy image of matrigel assay, showing that ANGPT2/LIF knockdown promotes tube formation of HUVECs. The scale bar represents 200 μm. *F*–*H*, statistical representation of matrigel assay, revealing that ANGPT2/LIF knockdown significantly increases the number of branches (*F*), the number of tubes (*G*), and the length of tubes (*H*) in the tubular structures compared with the control group. ∗∗*p* < 0.01, ∗∗∗*p* < 0.005 *versus* control, assessed using one-way ANOVA, n = 3. *I*, statistical plot of qPCR for detecting changes in VEGFa expression after miR-30a-5p overexpression. ∗∗*p* < 0.01 *versus* Mimic NC, assessed using Student's *t* test, n = 3. *J*, changes in VEGFa, LIFr, and TEK gene expression observed in sequencing after miR-30a-5p overexpression. ∗*p* < 0.05 *versus* Mimic NC, assessed by Student's *t* test, n = 3. Data are expressed as mean ± SEM. ANGPT2, angiopoietin-2; CCK8, Cell Counting Kit-8; HUVEC, human umbilical vein endothelial cell; qPCR, quantitative PCR; VEGF, vascular endothelial growth factor.

LIF and ANGPT2 can inhibit angiogenesis *in vivo* (30, 33). To further validate the role of ANGPT2 and LIF in HUVECs, siANGPT2 and siLIF were used to transfect HUVECs. The expression levels of ANGPT2 and LIF significantly decreased after transfection with siANGPT2 and siLIF (Fig. 6*A*). Compared to the control group, the cell viability of the siLIF group significantly increased (Fig. 6*B*). The cell migration assay showed that the migration rate in the siANGPT2 and siLIF groups significantly increased compared to the siNC group (Fig. 6, *C* and *D*). Angiogenesis assay results indicated significant increases in the number of branches, tube count, and tube length in the siANGPT2 and siLIF groups compared to the control group (Fig. 6, *E*–*H*). In summary, the downregulation of ANGPT2/LIF promoted tube formation in HUVECs. Notably, ANGPT2 only inhibits angiogenesis in the absence of vascular endothelial growth factor (VEGF) (34). Expression profiles of VEGFa, ANGPT2, and its receptor TIE-2 (TEK) were examined *in vitro* and *in vivo*. Results showed that VEGFa in the miR-30a-5p mimic group was significantly downregulated compared to the Mimic NC group (Fig. 6, *I* and *J*), consistent with corresponding transcriptome sequencing results and existing reports. Additionally, reduced VEGFa expression in TENG was observed after treatment with SKP-SC-EVs or miR-30a-5p (Fig. S3*B*).

## Discussion

EVs have garnered considerable attention as a novel approach for treating PNI (35). While SCs are preferred for supporting peripheral nerves, obtaining sufficient EVs from primary cultures is challenging due to their limited availability. In this study, SKP-SC-EVs were easily obtained and demonstrated *in vitro* proliferation. SKP-SCs share morphological and biological characteristics with primary cultured SCs, making them suitable donor cells. The potential of SKP-SC-EVs in repairing PNIs has been confirmed. Facilitating intercellular communication is essential for HUVECs to internalize EVs. SKP-SC-EVs were labeled with the lipid dye PKH67, and fluorescence signals of PKH67 in the cytoplasm and nucleus of HUVECs after EV treatment indicated successful internalization and functionality.

Although the complete composition of EVs remains undefined, they transport RNA, proteins, and lipids into cells (36). EVs can deliver miRNAs, inducing proangiogenic or antiangiogenic signaling (37). The noncoding RNA within SKP-SC-EVs presents a promising avenue for exploring mechanisms in treating PNIs. Angiogenesis is as crucial as axon regeneration in peripheral nerve regeneration (38). Genes regulating angiogenesis are already active on the first day of PNI (12). This study validated the highly expressed microRNA in SKP-SC-EVs using qRT-PCR, selecting fourteen microRNAs and identifying four with positive effects on angiogenesis through cell migration experiments. Previous research has shown that microRNAs enriched in EVs can promote sciatic nerve repair by stimulating axonal regeneration and SC myelination (29). This study established that SKP-SC-EVs repair PNI by promoting angiogenesis. Our research indicates that miR-30a-5p is one crucial miRNA by which SKP-SC-EVs promote angiogenesis. Some miRNAs, such as miR-21-5p, miR-21-5p, and miR-30a-5p, detected in SKP-SC-EVs might also have similar effects, while miR-30a-5p might played a significant role. SKP-SC-EVs significantly enhanced HUVEC viability, migration, and tube formation ability, and this effect could be inhibited entirely by miR-30a-5p inhibitor *in vitro*.

The expression of miR-30a-5p in HUVECs significantly increased after EV treatment, corresponding to its high enrichment in EVs. miR-30a-5p binds to the 3′-UTR of ANGPT2 and LIF, and its transfection led to a significant reduction in the expression of these genes *in vitro*. These findings suggest that miR-30a-5p in SKP-SC-EVs promotes angiogenesis by targeting ANGPT2 and LIF, providing a basis for understanding the molecular mechanisms of SKP-SC-EVs in treating PNI.

**Figure 7:**
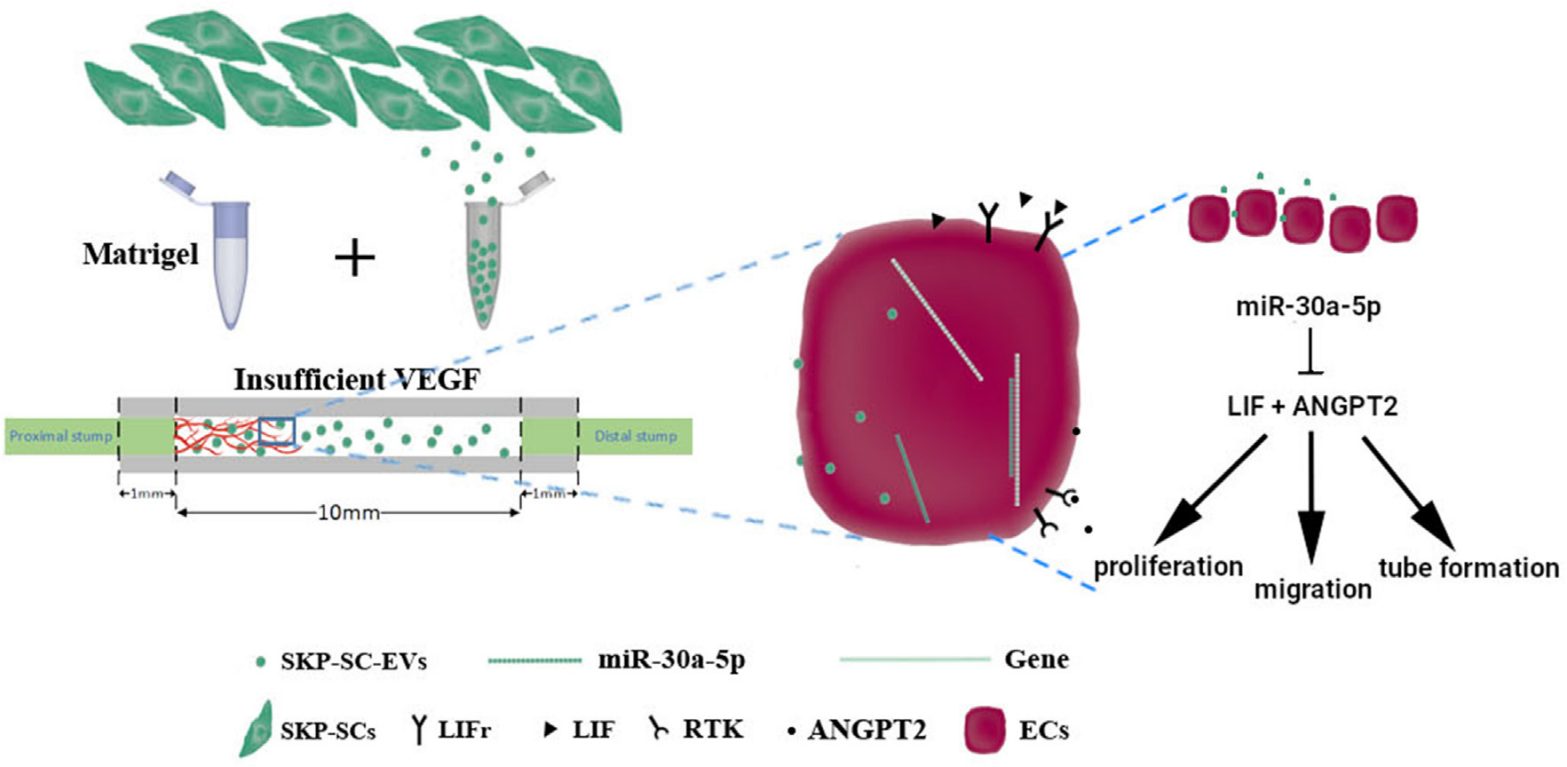
**Schematic drawing illustrating the mechanism of SKP-SC-EVs in promoting angiogenesis during peripheral nerve injury repair**. SKP-SC-EVs facilitate angiogenesis in the repair of rat sciatic nerve defects and enhance HUVEC vitality, migration, and tubulogenesis *in vitro*. Specifically, SKP-SC-EVs-derived miRNA-30a-5p promotes angiogenesis in peripheral nerve regeneration by targeting LIF and ANGPT2. SKP-SC-EVs, extracellular vesicles from skin precursor-derived Schwann cells; HUVEC, human umbilical vein endothelial cell; ANGPT2, angiopoietin-2.

Angiopoietin-2 (ANGPT2 or ANG2) belongs to the angiopoietin family, including ligands ANG1, ANG2, and ANG4, and receptors TIE-1 and TIE-2 (TEK). GO annotations link ANG2 to angiogenesis, where it functions bidirectionally; without VEGF, ANG2 may induce vascular degeneration by promoting endothelial cell apoptosis, while in the presence of VEGF, ANG2 can enhance capillary dilation, basement membrane remodeling, and endothelial cell proliferation and migration, stimulating new blood vessel growth. VEGF can convert ANG2's role from inhibiting to promoting angiogenesis (34, 39, 40). Plasmacytoma variant translocation 1 (lncRNA) promotes angiogenesis by binding and degrading miR-26b, which targets connective tissue growth factor and ANG2. In studies using the same cell line and medium but without supplemental VEGF, plasmacytoma variant translocation 1 demonstrated this effect (41). Consequently, miR-30a-5p targeting the 3′-UTR of ANG2 promoted HUVEC migration and tubule formation. Transcriptome sequencing and qRT-PCR of miR-30a-5p-transfected HUVECs showed decreased VEGF expression, elucidating the inhibitory effect of ANGPT2 on angiogenesis in this study. A working model (Fig. 7) illustrates that SKP-SC-EVs in TENG release miR-30a-5p, inhibiting ANGPT2 and LIF in the absence of VEGF.

Vascularization remains a significant challenge in the clinical application of tissue engineering products and technologies (42). The degree of vascularization of tissue-engineered nerve grafts *in vivo* directly influences the repair efficacy of surrounding nerves. Recent research has focused on scaffold material modification, seed cells, autologous vascular implantation, and angiogenesis-promoting factors. However, *in vitro* simulation of complex natural vascular systems presents considerable challenges, necessitating further research into biomaterials and engineering technologies (43). Prevascularization by implanting grafts into the body requires two surgeries, prolonging preoperative preparation and increasing complication risks. Direct combination with mature blood vessels also poses issues, including extended preoperative times and potential donor strain. Grafts based on endothelial cells or proangiogenic factors have addressed some of these problems, yet the integration of cell number, spatial distribution, concentration and release of loaded factors, mechanical properties, and compatibility of filling/scaffold materials require comprehensive consideration (44). Therefore, developing an efficient and feasible method to promote the vascularization of tissue-engineered nerve grafts is crucial for their widespread clinical application in treating peripheral nerve injuries. This study found that SKP-SC-EVs promote angiogenesis and nerve repair through miR-30a-5p. SKPs, isolated from human skin, are abundant and easily obtainable (45). EVs can be produced and modified *in vitro*, providing an experimental basis for clinical research on constructing tissue-engineered nerves conducive to angiogenesis.

Interleukin-6 family cytokines (LIF) encode a pleiotropic cytokine associated with vascular remodeling according to GO annotations. Studies on angiogenesis regulation have produced conflicting results. Researchers have noted that the concentration of LIF required for inhibition is significantly higher than that needed for promotion. Additionally, LIF exhibits highly variable effects on different subsets of endothelial cells, with responses varying across organs and developmental stages. These contradictory findings may be attributed to distinct signal transduction pathways and varying expression levels of cytokine receptors on endothelial cells (46, 47, 48). Similarly, a study involving miR-194 in liver-derived exosomes demonstrated its internalization by rat pulmonary microvascular endothelial cells, enhancing pulmonary microvascular endothelial cell proliferation, migration, and tubulogenesis by directly targeting the antiangiogenic genes THBS1, STAT1, and LIF (49). These findings support the present study's results, showing that miR-30a-5p promotes angiogenesis by targeting LIF.

While this study validated the mechanism by which miR-30a-5p promotes angiogenesis through targeting ANGPT2 and LIF, the specific downstream molecular pathways remain unclear. The roles of ANGPT2 and LIF, along with their associated signaling pathways, in peripheral nerve and vascular regeneration, require further exploration. Additionally, this study did not investigate other miRNAs that may enhance HUVEC migration. The therapeutic effects of SKP-SC-EVs in PNI treatment likely involve multiple miRNAs. Comprehensive studies on the structure, sequence, and target genes of various miRNAs with angiogenic properties may yield even more promising results.

## Experimental procedures

### Animals

A total of 392 male Sprague-Dawley rats, weighing 180 to 200 g, were provided by the Laboratory Animal Center of Nantong University. Animals were numbered based on their weight and assigned to control and experimental groups using a random number table. The rats were housed in polypropylene cages (5 rats per cage) with free access to food and water, maintained in a controlled environment (23 ± 2 °C, 40–65% humidity) with a 12-h light/dark cycle. All experimental protocols involving animals adhered to the Chinese Guidelines for the Care and Use of Laboratory Animals and were approved by the Administration Committee of Experimental Animals of Nantong University, China (approval No. S20210302–036).

### Nerve defect injury and treatment

Adult male Sprague-Dawley rats weighing 180 to 200 g were numbered by weight and randomly assigned to different groups using a random number table method. Anesthesia was induced *via* intraperitoneal injection of a compound anesthetic (chloral hydrate 4.25 g, magnesium sulfate 2.12 g, sodium pentobarbital 886 mg, ethanol 14.25 ml, and propylene glycol 33.8 ml in 100 ml) at a dose of 0.2 to 0.3 ml/100 g body weight. Upon achieving deep anesthesia, an incision was made on the skin of the right thigh, followed by blunt separation of the lateral muscles to expose the sciatic nerve. A 1 cm segment of the sciatic nerve was excised, creating a defect. Subsequently, vehicle or EVs mixed with Matrigel were loaded into a silicone conduit (inner diameter: 2.0 mm) and applied to bridge the nerve defects, as previously described (30). The rats were placed on a warming blanket maintained at 37 °C until fully awake and then provided ibuprofen for pain relief. All animal experiments adhered to situational animal care guidelines and received ethical approval from the Administration Committee of Experimental Animals, Jiangsu Province, China.

### Cell culture

SKP-SCs were successfully isolated and induced from neonatal rats and cryopreserved for subsequent experiments as previously described (50, 51). Resuscitated cells were subcultured on plates coated with poly-D-lysine (Sigma-Aldrich) and laminin (Corning) in an SC proliferation medium consisting of Dulbecco's modified Eagle's medium/F12 (3:1) containing 1% penicillin/streptomycin, 2% N2 supplement (STEMCELL Technologies), 3% fetal bovine serum (FBS; Gibco), 5 μM forskolin (Sigma-Aldrich), and 50 ng/ml heregulin-1β (R&D). HUVECs were purchased from Cyagen and cultured in endothelial cell medium (ScienCell Research Laboratories) supplemented with 5% FBS, 1% endothelial cell growth supplement, and 100 U/ml penicillin/streptomycin.

### SKP-SC-EVs isolation and characterization

SKP-SCs were cultured in a serum-free proliferation medium for 48 h after reaching 80% confluency. The medium was collected and subjected to ultracentrifugation at 500g for 10 min. Cells and debris in the supernatant were removed using a 0.22-μm filter (Millipore). Following the manufacturer's instructions, EVs were extracted from the filtered supernatant using the exoEasy Kit (QIAGEN) and further concentrated with an Amicon Ultra 10 kDa tube (Millipore). Nanoparticle tracking analysis (German Particle Metrix) determined the diameter distribution and concentration of SKP-SC-EVs. A transmission electron microscope (Hitachi) was used to observe the morphology of EVs. Finally, Western blot analysis detected the expression of EV-associated markers, including Alix, CD9, CD63, CD81, Hsp70, and TSG101.

### Internalization of fluorescent dye-labeled SKP-SC-EVs by cells

According to the manufacturer's instructions, EVs were labeled with PKH67 (Sigma-Aldrich). Fluorescent dye-labeled EVs or the vehicle was then cocultured with HUVECs for 4 h. The cells were washed with PBS, fixed in 4% paraformaldehyde, and imaged using a fluorescence microscope (Axio Imager M2, Zeiss) after CD31 immunofluorescence staining. To assess EV internalization *in vivo*, 10 μl of fluorescent dye-labeled EVs or the vehicle was injected into the sciatic nerve epineurium. At 24 h post operation, a portion of the nerve was separated, dehydrated, frozen, sectioned using a cryostat, and stained with CD31 and secondary antibodies. Photomicrographs were captured using a fluorescence microscope.

### miRNA and mRNA sequencing

Total RNA was extracted using the mirVana miRNA Isolation Kit (Ambion) following the manufacturer's instructions (n = 10 per group). RNA quality was assessed with a BioAnalyzer 2100 (Agilent Technologies), and quantified by absorbance at 260 nm using a NanoDrop 2000 spectrophotometer (Infinigen Biotechnology Inc). miRNA sequencing was performed at GENEWIZ Co., Ltd and OE Biotech Co., Ltd. mRNA sequencing was conducted at GENE DENOVO Co., Ltd. Sequencing data were deposited in the NCBI database under GEO accession number GSE245328. Predicted target genes were identified using Hybrid and miRWalk.

### Bioinformatics analysis

Differential expression analysis of RNA was conducted using DESeq2 software (http://www.bioconductor.org/packages/release/bioc/html/DESeq2.html) between two groups. Genes with a *p*-value below 0.05 and an absolute fold change of ≥2 were considered differentially expressed. Weighted gene coexpression network analysis analyzed gene expression patterns across three sample groups at 11 time points. The R language software package (http://cran.r-project.org/) was used to construct a gene coexpression network, clustering genes with similar expression patterns and assessing correlations between modules and specific traits or phenotypes. The analysis involved the following steps: (1) assuming a scale-free distribution for the gene network, defining the gene co-expression correlation matrix and the adjacency function; (2) calculating differentiation coefficients for different nodes and constructing a hierarchical clustering tree. Different branches of the clustering tree represented distinct gene modules with high coexpression within the modules; (3) exploring correlations between modules and specific biological processes, and comparing dynamic changes in axon regeneration, myelination, angiogenesis, inflammatory chemotaxis, cell death, proliferation, and migration involved in the regeneration microenvironment among the three groups during nerve defect repair.

### Immunofluorescence staining

At room temperature (RT), HUVECs or tissue sections were fixed with 4% paraformaldehyde (Beyotime) and blocked with a blocking solution (Beyotime). Primary antibodies were incubated with the samples overnight at 4 °C for immunofluorescence staining, followed by secondary antibody incubation at RT in the dark. Nuclei were counterstained with Hoechst 33342 (1:1000 dilution; Abcam). Primary antibodies included goat anti-CD31 (5–15 μg/ml, R&D) and mouse anti-alpha smooth muscle actin (1:150 dilution; Abcam). Fluorescent-labeled secondary antibodies used were donkey anti-goat IgG cyanine 3 (Cy3; 1:400 dilution; Abcam) and donkey anti-mouse IgG 488 (1:400 dilution; Thermo Fisher Scientific). Photomicrographs were captured using a fluorescence or confocal microscope (SP5; Leica).

### Cell transfection assay

miRNA mimics and siRNA were obtained from RiboBio Co., Ltd and transfected into HUVECs using Lipofectamine RNAiMAX Reagent (Invitrogen), following the manufacturer's protocol. The detailed experimental steps followed the manufacturer’s protocol. The target sequences of siRNA were listed in Table 1. The mimic sequences of miRNA were listed in Table 2.

**Table 1:** The target sequences of siRNA

Target gene	NO.	Target sequence (5′-3′)
ANGPT2	siRNA-1	GCAACTGACTAATCAGCAA
ANGPT2	siRNA-2	GGAGACAGTTAATAACTTA
ANGPT2	siRNA-3	CAGAGACTGTGCTGAAGTA
LIF	siRNA-1	ACCGCATAGTCGTGTACCT
LIF	siRNA-2	GGGACCAGAAGATCCTCAA
LIF	siRNA-3	GCACGGAGAAGGCCAAGCT
Control	siRNA	GGCTCTAGAAAAGCCTATGC

**Table 2:** The sequences of miRNA

Gene	Mimic sequence (5′-3′)
rno-miR-30a-5p	UGUAAACAUCCUCGACUGGAAG
rno-miR-21-5p	UAGCUUAUCAGACUGAUGUUGA
rno-miR-22-3p	AAGCUGCCAGUUGAAGAACUGU
rno-miR-30b-5p	UGUAAACAUCCUACACUCAGCU

### Cell viability assay

To assess HUVEC cell viability following EV or miRNA mimic internalization, CCK8 (Dojindo) was mixed with the medium in a 10:1 ratio. The original culture medium was replaced, and cells were incubated at 37 °C for 4 h. Absorbance was measured at 450 nm using a microplate reader (BioTek). Cell viability was expressed as a percentage relative to the control group.

### Wound-healing assay

A wound-healing assay was used to evaluate the effect of EVs on HUVEC migration. Briefly, 1 × 10^4^ HUVECs were seeded into the wells of a culture insert (ibidi) and coincubated with varying concentrations of EVs in a medium with 0.25% FBS. miRNA-treated cells were seeded into the wells posttransfection. After 24 h, the culture insert was removed, and images were captured under a light microscope immediately and 12 h later. The migratory ratio was calculated to assess the migration ability of HUVECs.

### Tube formation assay

To evaluate the angiogenic potential of EVs, HUVECs were seeded at 1 × 10^4^ cells per well in a 96-well plate coated with 50 μl of Matrigel (BD Biosciences). Tubular structures were visualized using an optical microscope, and ImageJ software (National Institutes of Health, Bethesda, MD, USA; http://imagej.nih.gov/ij/) was used to measure tube length, number of tubes, and branch points.

### Angiogenesis assay

The angiogenesis assay of the nerve bridge was performed by perfusing it with contrast agents (Flow Tech, Inc) following protocol (52). Eight weeks post surgery, the rats were deeply anesthetized and infused with approximately 500 ml of normal saline mixed with 0.8 ml of heparin sodium (Changzhou Qianhong Pharmaceutical Co., Ltd). The blue-colored (MV120) MICROFIL compound (20 ml of MV, 25 ml of diluent, and 2.25 ml of curing agent) was infused into the aorta using a 5-mL syringe within 20 min of mixing. Each animal received approximately 40 ml of the contrast agent. After ligating the aorta root with surgical sutures, the rats were housed at 4 °C overnight for curing. Finally, the tissue-engineered nerve and nerve ends were harvested and cleared in glycerin. The samples were incubated in a mixture of water and glycerin, with the glycerin concentration gradually increased to 75%, 85%, and 100% at successive 24-h intervals until the samples became transparent.

### Luciferase reporter assay

The 3′-UTR sequences of ANGPT2 and LIF were amplified from genomic DNA and subcloned downstream of the luciferase gene stop codon in the luciferase reporter vector (53). Using appropriate primers, PCR amplification of the 3′-UTR sequences generated distinct p-Luc-UTR luciferase reporter vectors. Sequencing verified both WT and mutant 3′-UTR sequences. HEK 293T cells were seeded in a 96-well plate and transfected with a mixture of 30 ng of p-Luc-UTR, 5 pmol of miRNA mimic, and 5 ng of Renilla luciferase, according to the Lipofectamine 3000 transfection system protocol (Invitrogen). After 48 h of incubation, firefly and *Renilla* luciferase activities were measured in cell lysates using the dual-luciferase reporter assay system (Promega).

### Plantar test assay

The plantar test was conducted 6 months after catheter transplantation to assess sensory function recovery in the injured hind limb. iodophor and picric acid were used to prevent toe-biting behavior in rats. The rats were acclimatized in separate plexiglass boxes for at least 30 min before testing. Radiant heat flux was applied to the right hind paw through the glass, and the withdrawal response time was recorded. If the rat did not retract its paw within 30 s, the thermal stimulation automatically ceased to prevent thermal damage. The experiment was repeated three times with a 10-min interval between each test.

### Electrophysiological assessment

Six months post operation, an electrophysiological examination was conducted following established protocols (54, 55). CMAP) was recorded using electromyography and an evoked potentiometer (Keypoint, Alpine BioMed ApS). Electrical stimulation was applied to the distal and proximal sciatic nerves at the injury site using a bipolar hook electrode and a unipolar needle. A recording electrode was placed in the ipsilateral ventral gastrocnemius muscle, and a ground electrode was inserted into the dorsal skin. A supramaximal stimulus (1 mA) was used to elicit the maximum CMAP response. Recordings were repeated three times, with amplitude or delay averaging the CMAP onset time for each rat. Motor nerve conduction velocity was calculated by dividing the distance between the proximal and distal stimulation sites by the latency difference. The experiment was conducted at RT, with rats maintained at 37 °C using a thermal blanket.

### Ultrasound imaging analysis

Six months post surgery, the rats were anesthetized and depilated. Blood flow in the hind limbs was monitored using a laser Doppler perfusion imager (PeriScan PIM 3 System, Perimed AB). The perfusion ratio was determined from ultrasound images, expressed as the ratio of the ischemic area to the contralateral nonischemic area on the surgical side.

### Quantitative reverse transcription–polymerase chain reaction

Total RNA was extracted from cells or nervous tissue and subjected to reverse transcription to obtain complementary DNA using the Omniscript RT kit (QIAGEN). qRT-PCR was then performed with SYBR Premix (QIAGEN) on the BIO-RAD system (BIO-RAD-96CFX) following standard methods. miRNA primers were purchased from RiboBio, and U6 expression was used for normalization. All procedures were conducted in strict accordance with the manufacturer's instructions.

### Western blotting

Proteins were extracted from EVs or cells using Pierce RIPA Lysis Buffer (Thermo Fisher Scientific) containing 1 mM PMSF (Sigma Chem) and protease and phosphatase inhibitors (Cell Signaling Technology). Following centrifugation, protein concentration was quantified using a bicinchoninic acid protein assay kit (Beyotime) according to the manufacturer's protocol. Total proteins were separated by 10%-12% SDS-PAGE and transferred onto polyvinylidene difluoride membranes (Millipore). Membranes were blocked with 5% nonfat milk in Tris-HCl–buffered saline for 1 h at RT and incubated with primary antibodies overnight at 4 °C. Primary antibodies included heat shock protein 70 (HSP70; 1:1000; Proteintech), Alix (1:1000; Abcam), CD63 (1:1000; Abcam), CD9 (1:1000; Abcam), CD81 (1:1000; Sigma-Aldrich), tumor susceptibility gene 101 (TSG101; 1:1000; Proteintech), ANGPT2 (1:1000; Abcam), and LIF (1:1000; Abcam). Horseradish peroxidase–labeled goat anti-mouse IgG (1:5000; Santa Cruz Biotechnology) and horseradish peroxidase-labeled chicken antirabbit IgG (1:5000; Abcam) were applied at RT for 1 h. Signals on the membranes were detected using enhanced chemiluminescence reagent kits (Beyotime) and conjugated to the membranes at RT for 1 h.

### Statistical analysis

Data analysis was performed using GraphPad software 8.0 (https://www.graphpad.com/), based on at least three independent experiments. Results were presented as mean ± standard error of the mean. The Student's *t* test was used for comparisons between two groups, while ANOVA followed by Tukey's test was applied for multiple comparisons.

## Conclusion

In summary, this study demonstrated that SKP-SC-EVs effectively promote angiogenesis in repairing sciatic nerve defects in rats. It further confirmed the enhancement of HUVEC viability, migration, and tube formation *in vitro*. miR-30a-5p, along with its target genes ANGPT2 and LIF, plays a pivotal role in the function of SKP-SC-EVs (Fig. 7). This research provides both theoretical and experimental foundations for developing cell-free tissue-engineered nerves in PNI treatment and unveils a new molecular target for clinical therapy.

## Data availability

The transcriptome sequencing data have been deposited in the NCBI database with the GEO accession number GSE245328. The manuscript and supplemental materials contain all the data collected during the current study, and the corresponding author can provide unprocessed data upon reasonable request.

## Supporting information

This article contains supporting information.

## Conflict of interest

The authors declare that they have no conflicts of interest with the contents of this article.

## Figures and Tables

**Fig. S3:**