# Supplementary material for: Angiogenesis-promoting effect of SKP-SC-EVs-derived miRNA-30a-5p in peripheral nerve regeneration by targeting LIF and ANGPT2
Source: J Biol Chem. 2024 Dec 26;301(2):108146. doi: 10.1016/j.jbc.2024.108146 (PMC11791313; doi:10.1016/j.jbc.2024.108146)
Supplement: Supplementary Figure legends [file mmc1.docx]

**Supplementary Figure legends:**

**Figure S1. Characterizing SKP-SC-EVs in vitro and in vivo. A**, Immunofluorescence analysis revealed that SKP-SCs displayed the characteristic bipolar spindle shape, aligned side by side, and expressed SC-related markers S100 (red) and GFAP (green). Nuclei were labeled with DAPI (blue). Scale bar: 50 μm. **B**, Transmission electron microscopy (TEM) depicted the distinctive sunken cup-like structure of EVs. Scale bar: 200 nm. **C**, Representative image displaying the size distribution of EVs as detected by nanoparticle tracking analysis (NTA). **D**, Western blot analysis of EV protein expression. Compared with the source cells, Alix, CD9, CD63, CD81, Hsp70, and TSG101 were highly expressed in EVs, while the expression of ER protein Calnexin was low in SKP-SC-EVs. These results affirm the suitability of SKP-SC-EVs for subsequent experiments. **E**, Internalization of EVs in the sciatic nerve. EVs were labeled with PKH26 (red), blood vessels were marked with CD31 (green), and nuclei were stained with DAPI (blue). Scale bar: 1000 μm. **F**, Comparison of the tibialis anterior, gastrocnemius, and soleus muscles between normal and injured conditions. **G**, Wet weight ratio of muscle. n = 3, assessed using a one-way analysis of variance. Data are expressed as mean ± SEM.

**Figure S2. miR-30a-5p inhibitor attenuates the pro-migratory effect of SKP-SC-EVs. A**, Light microscopy image of wound healing demonstrating that knockdown of miR-30a-5p inhibits the pro-migration effect of SKP-SC-EVs. Scale bar: 200 μm. **B**, Statistical graph of wound healing, indicating that knockdown of miR-30a-5p dampens the pro-migration effect of SKP-SC-EVs. n=3, assessed by one-way analysis of variance. **P*<0.05, n.s. *P*>0.05 vs control. Data are expressed as mean ± SEM.

**Figure S3. Pro-angiogenic role of SKP-SC-EVs in peripheral nerve regeneration. A**, Transcriptome sequencing results highlighting the regulation of KEGG biological processes. The most enriched pathways included cytokine-cytokine receptor interaction, cell cycle, cell adhesion molecules, cell senescence, Jak-Stat signaling pathway, and vascular smooth muscle contraction. **B**, Immunofluorescence analysis revealed a decrease in VEGFa expression in TENG after treatment with SKP-SC-EVs. VEGFa, marking vascular endothelial growth factor, is shown in red. The expression of VEGFa decreased following SKP-SC-EVs treatment. Full image Scale bar: 200 μm; Magnified view Scale bar: 50 μm.
